# Supplementary material for: Frequent 4EBP1 Amplification Induces Synthetic Dependence on FGFR Signaling in Cancer
Source: Cancers (Basel). 2022 May 13;14(10):2397. doi: 10.3390/cancers14102397 (PMC9139685; doi:10.3390/cancers14102397)
Supplement: Supplementary file 1 [file cancers-14-02397-s001.zip › Figures S1-S5.pdf]

Supplementary Figures

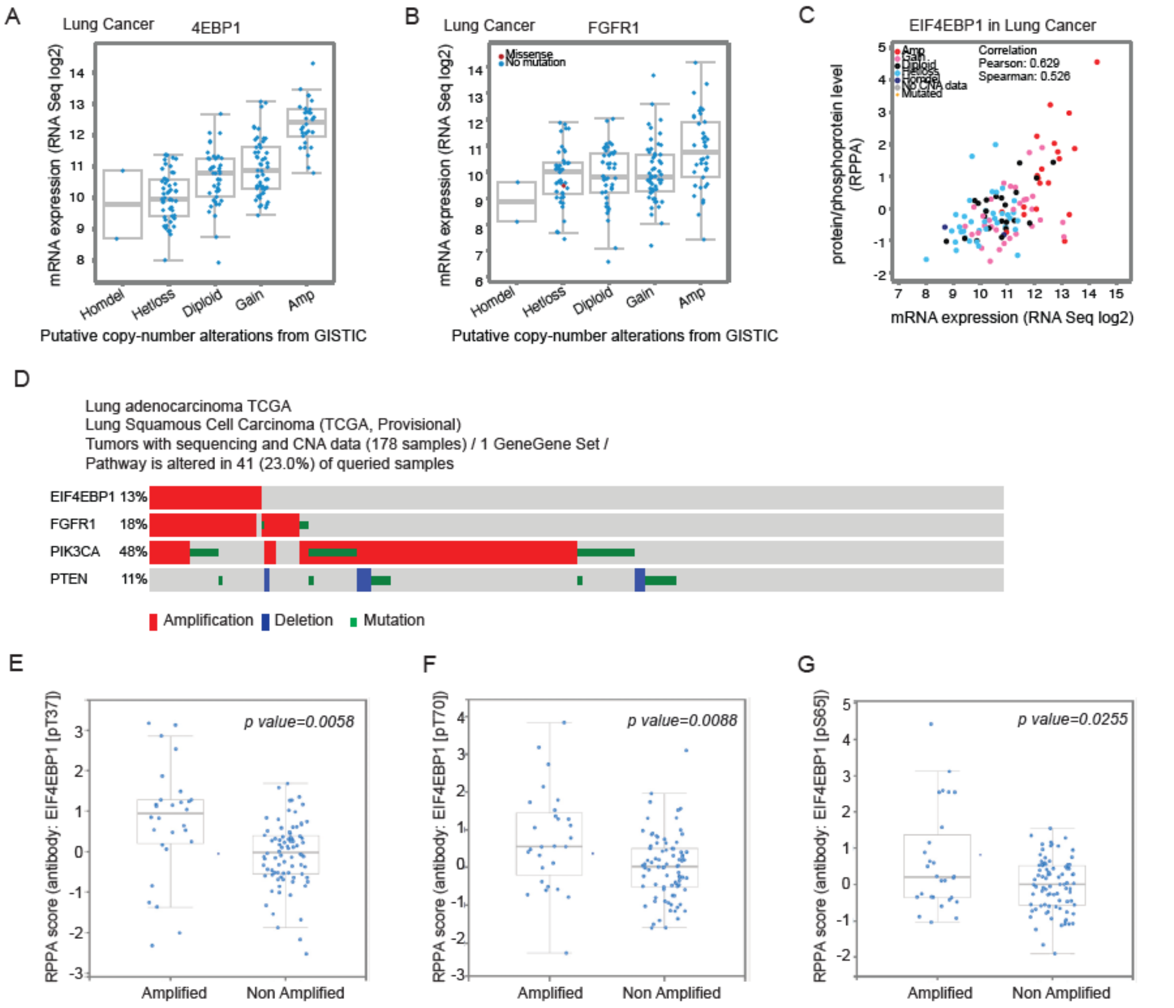

Figure S1. 4EBP1 is a target of genomic copy number gains (Chr. 8p11) in breast and lung cancer. A. Correlation plots showing positive correlation of 4EBP1 and FGFR1 copy number in breast cancer patient samples (TCGA and cBio portal data from MSKCC). **B and C.** Correlation plots showing positive correlation of mRNA expression with copy number of 4EBP1 and FGFR1 in lung cancer patient samples (TCGA and cbio portal data from MSKCC). **D.** 4EBP1 protein expression is elevated and correlated positively with the mRNA levels in 4EBP1 amplified lung cancer patient samples. **E.** Oncoprint map showing the frequency of amplification of 4EBP1, FGFR1, PI3K, and PTEN in lung cancer (TCGA and cbio portal data from MSKCC). **F-H.** Reverse Phase Protein Array (RPPA) analysis shows phospho-4EBP1 (T37, T70, and S65) protein is upregulated in 4EBP1 and FGFR1 amplified lung cancer patients.

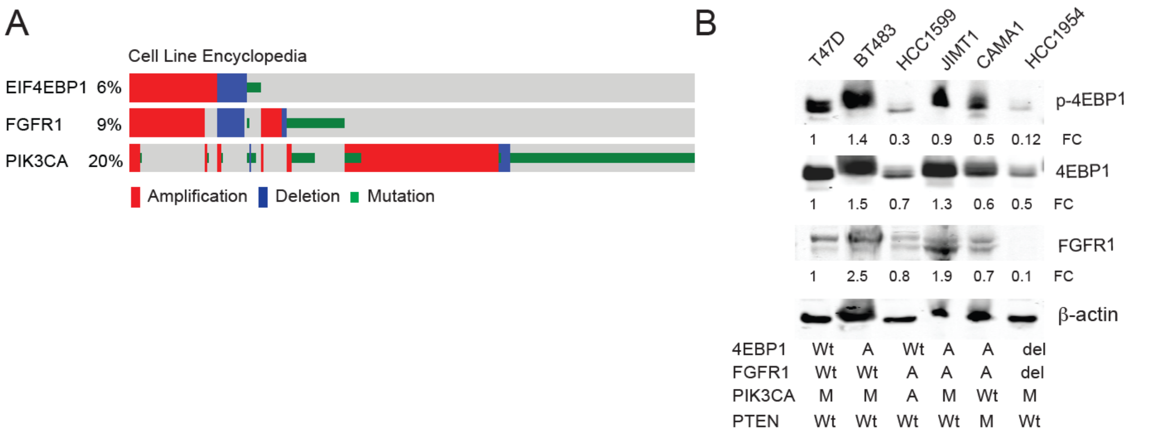

Figure S2. Loss of 4EBP1 results in activated translation and reduced sensitivity to FGFR1 and PI3K inhibition. A. Oncoprint map showing the frequency of amplification of 4EBP1, FGFR1, and PI3KCA, in Cell Line Encyclopedia data. **B.** Immunoblot analysis showing 4EBP1, p-4EBP1 (Ser 65), and FGFR1 levels in breast cancer cell lines with wild type or amplified 4EBP1. B-actin is used as loading control.

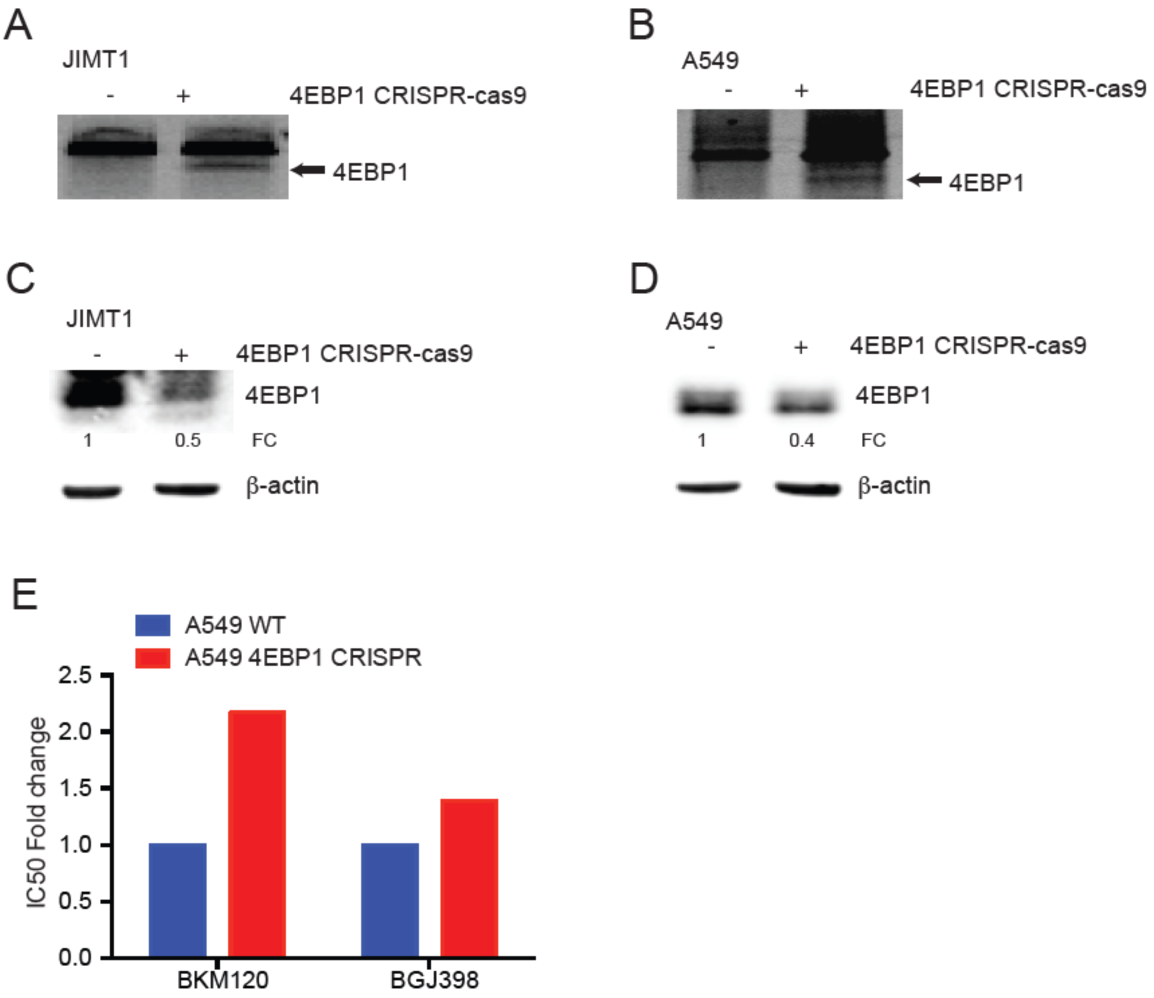

Figure S3. 4EBP1 Amplified tumors show increased sensitivity to FGFR1 and PI3K inhibition. A and B. Surveyor Nuclease assay showing CRIPSR-cas9 edited 4EBP1 in JIMT1 **(A)** and A549 **(B)** cells. **C and D.** Immunoblot showing downregulation of 4EBP1 in 4EBP1-CRISPR-cas9 edited JIMT1 **(C)** and A549 **(D)** cells. **E.** Cell viability assay showing that IC50 is increased in A549 4EBP1-

CRISPR deleted cells compared to wild type cells in response to BGJ398 and BKM120.

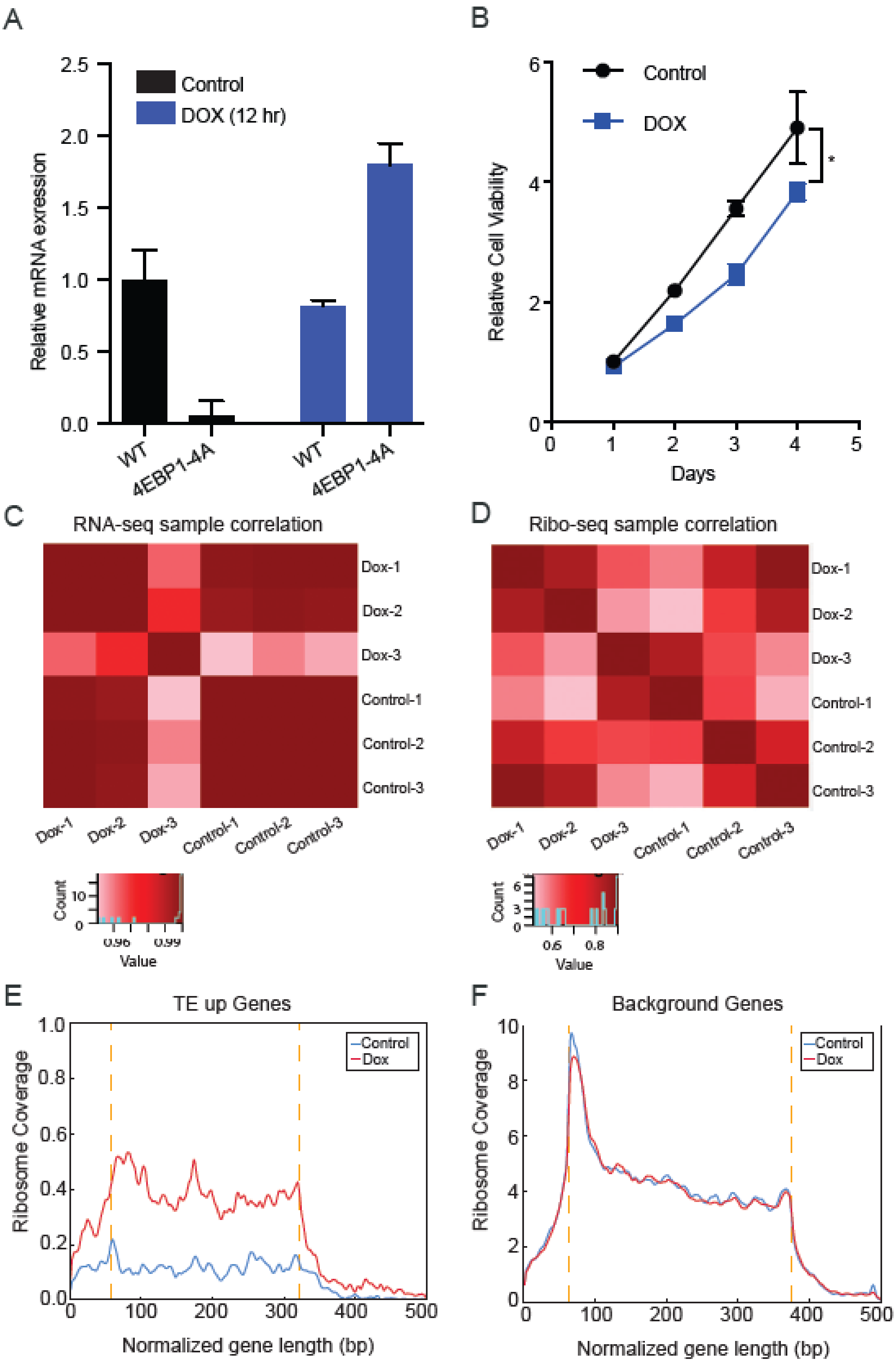

Figure S4. Ribosome foot printing identifies translational targets of 4EBP1. A. RNA expression analysis of 4EBP1 in 293T cells transduced with 4EBP1-4A showing induction of 4EBP1-4A mRNA following doxycycline (2 mg/ml at 12

hr) treatment. **B.** Cell viability in 293T cells transduced with 4EBP1-4A following DMSO or doxycycline treatment (2 mg/ml) showing reduction in cell viability in 4EBP1-4A expressing cells at the indicated time points. **C and D.** Read counts correlation plots of replicates from control and doxycycline treated total RNA **(C)** and ribosome foot printing **(D)** samples. **E and F.** Ribosome coverage is increased throughout the mRNA length in the TE up mRNAs **(E)** while remains unchanged in background mRNAs **(F)** in doxycycline treated cells compared to the control cells.

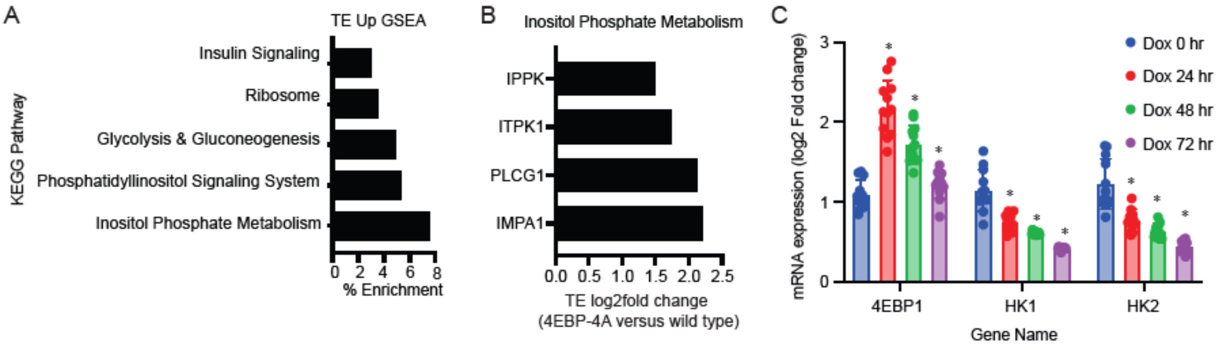

Figure S5. 4EBP1 controls translation of genes involved in insulin signaling, glucose metabolism, and inositol pathway. A. GSEA KEGG pathway analysis of 4EBP1 dependent (TE down) genes. **B.** TE (log2fold change) of key genes involved in Inositol phosphate metabolism. **C.** RNA expression analysis of 4EBP1, HK1, and HK2 in 293T cells transduced with 4EBP1-4A showing induction of 4EBP1-4A mRNA following doxycycline (2 mg/ml at 12 hr) treatment.
