# Supplementary figures and images for: Frequent 4EBP1 Amplification Induces Synthetic Dependence on FGFR Signaling in Cancer
Source: Cancers (Basel). 2022 May 13;14(10):2397. doi: 10.3390/cancers14102397 (PMC9139685; doi:10.3390/cancers14102397)

Supplementary Figure 6  
Fig. 2A and 2B

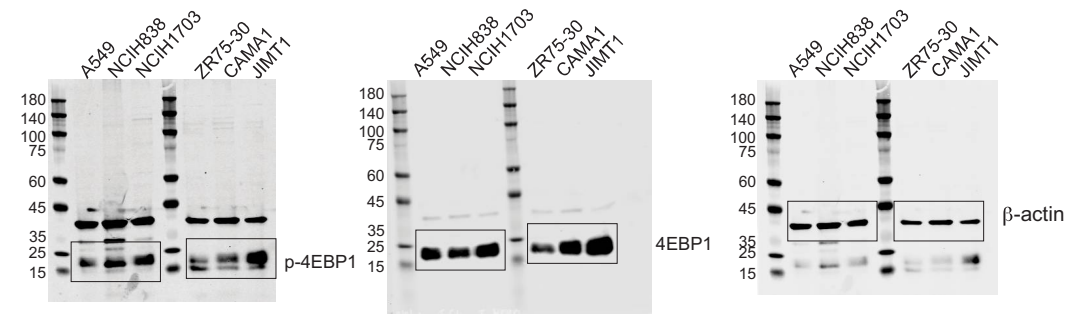

Supplementary Fig. 2B

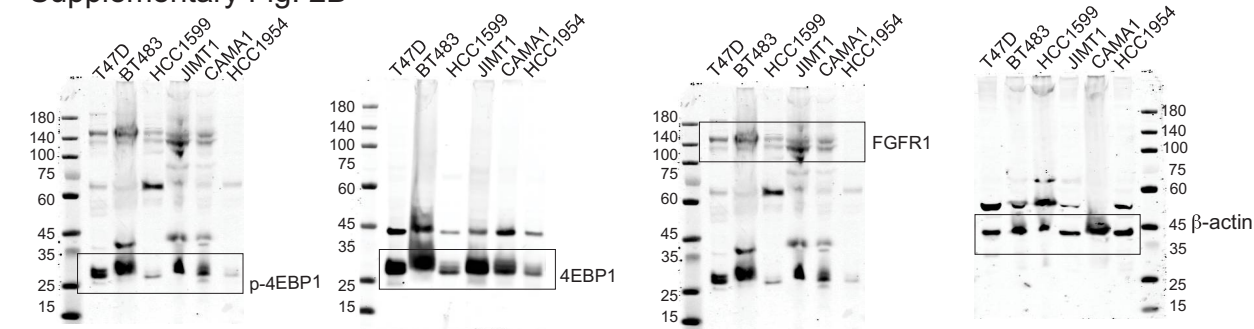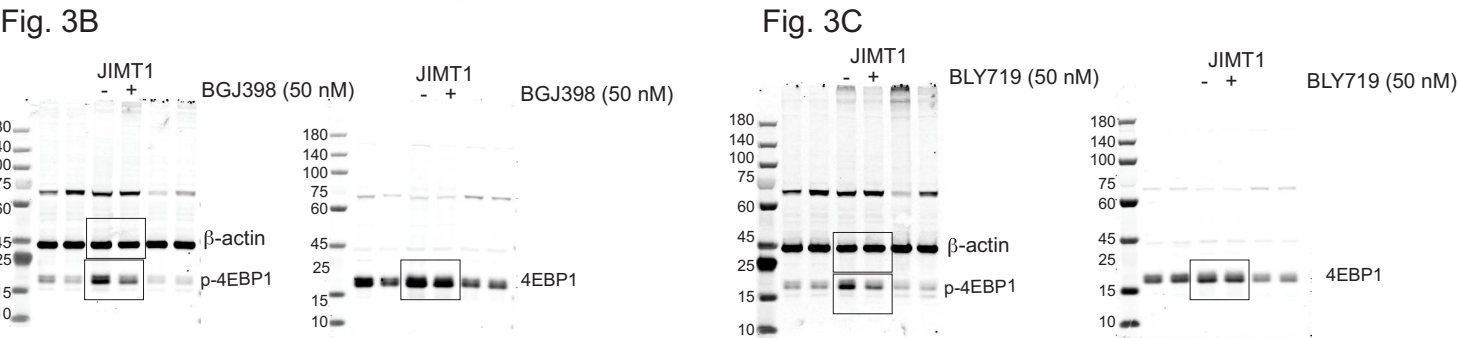

Supplementary Figure 3C and 3D

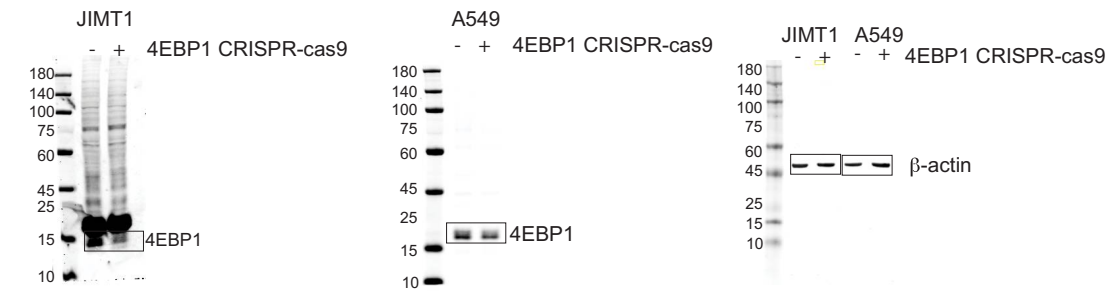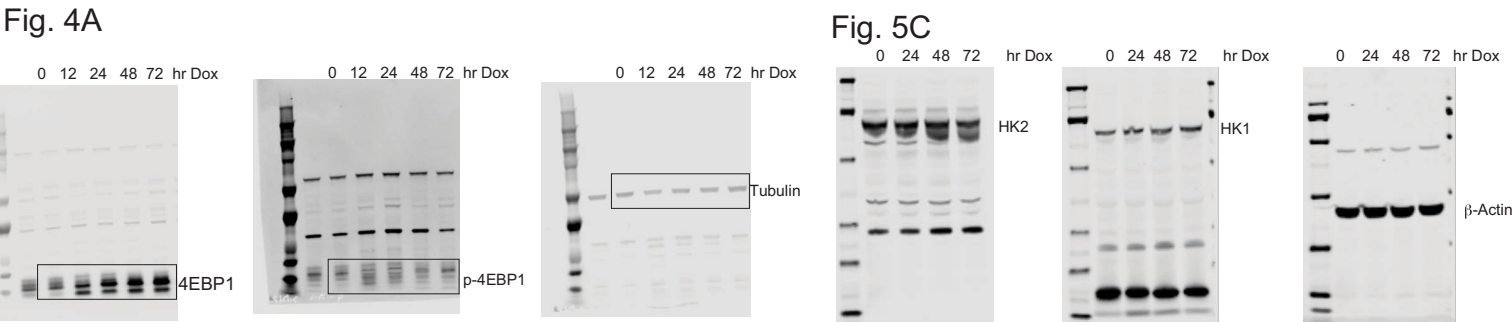

Supplement: Supplementary file 1 [file cancers-14-02397-s001.zip › Figures S6.pdf]
